# White Matter Integrity Pre- and Post Marijuana and Alcohol Initiation in Adolescence 

**DOI:** 10.3390/brainsci3010396

**Published:** 2013-03-22

**Authors:** Joanna Jacobus, Lindsay M. Squeglia, M. Alejandra Infante, Sunita Bava, Susan F. Tapert

**Affiliations:** 1 VA San Diego Healthcare System Psychology Service (116B), 3350 La Jolla Village Drive, San Diego, CA 92126, USA; E-Mails: jjacobus@ucsd.edu (J.J.); sbava@ucsd.edu (S.B.); 2 Department of Psychiatry, University of California, San Diego, 9500 Gilman Drive (0603), La Jolla, CA 92093, USA; E-Mails: lsquegli@ucsd.edu (L.M.S.); minfante@ucsd.edu (M.A.I.); 3 San Diego Joint Doctoral Program in Clinical Psychology, San Diego State University/University of California, 6363 Alvarado Court, Suite 103, San Diego, CA 92120, USA

**Keywords:** adolescence, alcohol, marijuana, white matter, diffusion tensor imaging, cognition, development, brain

## Abstract

Characterizing the effects of alcohol and marijuana use on adolescent brain development is important for understanding potential alterations in neurodevelopment. Several cross sectional studies have identified group differences in white matter integrity after initiation of heavy alcohol and marijuana use, however none have explored white matter trajectories in adolescents pre- and post initiation of use, particularly for marijuana users. This study followed 16 adolescents with minimal alcohol and marijuana use at ages 16–18 over three years. At follow-up, teens were 19–22 years old; half of the participants initiated heavy alcohol use and half initiated heavy alcohol *and* marijuana use. Repeated-measures ANOVA revealed 20 clusters in association and projection fibers tracts (*p* < 0.01) in which a group by time interaction was found. Most consistently, white matter integrity (*i.e.*, fractional anisotropy) decreased for those who initiated both heavy alcohol and marijuana use over the follow-up interval. No effect of time or change in white matter integrity was seen for those who initiated alcohol use only in the majority of clusters. In most regions, at the baseline time point, teens who would later initiate both alcohol and marijuana use demonstrated white matter integrity greater than or equal to teens that initiated alcohol use only. Findings suggest poorer tissue integrity associated with combined initiation of heavy alcohol and marijuana use in late adolescence. While pre-existing differences may also be related to likelihood of substance use, the present data suggest an effect on tissue integrity for these teens transitioning to combined alcohol and marijuana use in later adolescence.

## 1. Introduction

Alcohol and marijuana use are typically initiated during adolescence, with rates of lifetime use increasing significantly into young adulthood. Alcohol use increases from 30% to 70% between 8th and 12th grade, with 24% of high school seniors endorsing recent binge drinking (*i.e.*, >5 drinks in a row in the last two weeks). Marijuana, the second most commonly used substance, shows similar increases with rates of use increasing from 15% to 45% during the same time period [1]. In fact, perceived risk of marijuana use has been declining, and the percent of high school seniors identifying as daily users (7%) has generally increased over the past two decades. 

While alcohol and marijuana are the most commonly used substances among adolescents, their potential effects on the developing brain are not well understood. Elucidating the unique and interactive effects of these substances on brain development is crucial, as significant neural maturation in both gray and white matter occurs during adolescence. Specifically, the adolescent brain undergoes marked decreases in gray matter along with linear increases in white matter, purportedly associated with myelination and to some degree, regressive changes to axons and dendrites [2,3,4]. Progressive myelination during adolescence is likely associated with optimal cognitive processing and communication between brain regions [5], which in turn may result in better cognitive performance [6,7,8]. Any potential neurotoxic insults during this crucial maturation process could have long-lasting implications on cognitive development [9,10,11]. 

Longitudinal studies have found that initiation of alcohol use alone during adolescence is associated with poorer neuropsychological functioning over time, including worsening visuospatial functioning and attention [12,13,14] as well as deviations in brain response patterns when compared to non-using adolescent controls [15,16]. Widespread reductions in white matter integrity have also been found in adolescent binge drinkers when compared to non-drinking teenagers [17]. 

Adolescents who use marijuana typically engage in alcohol use [1]; therefore, it is not surprising that much of the marijuana-focused literature includes adolescents who use both marijuana and alcohol to some extent. Cross-sectional neuropsychological findings show that adolescent heavy marijuana users have worse psychomotor speed, complex attention, story memory, and planning and sequencing abilities, even after a month of abstinence, as well as deficits on tests of verbal and visual memory when compared to non-users [18,19,20]. Longitudinal examinations have found poorer verbal and working memory, which remitted with abstinence, but continued attention deficits [21]. Initiation of marijuana use during adolescence may have long-lasting neurocognitive consequences [22], particularly in the areas of sustained attention, executive functioning [23], impulse control [24], and verbal memory [19].

Structural and functional imaging has helped better elucidate the potential neurobiological underpinnings of these cognitive deficits. The only existing longitudinal imaging study reporting pre-and post marijuana use data found that smaller orbitofrontal volumes at age 12 predicted marijuana initiation by age 16 [25]. Smaller medial orbital frontal cortex morphometry has also been identified in adolescent marijuana users ages 16–19, and linked with younger age of initiation [26]. Cross-sectional structural MRI studies have found thinner cortices in prefrontal and insular regions and thicker cortices in posterior regions [27] in marijuana using adolescents compared to healthy controls; however it is unclear if differences existed prior to marijuana use. Several functional imaging studies have shown aberrations in brain response patterns and cerebral blood flow in marijuana and alcohol using adolescents [28,29,30,31,32,33]. There is some suggestion that these differences remit with abstinence [28,29], however findings are mixed [30,34]. 

Diffusion tensor imaging (DTI) studies have been fairly consistent in regards to white matter integrity in comorbid marijuana and alcohol using adolescents, with most cross-sectional research showing poorer white matter integrity (e.g., *decreased* fractional anisotropy) in alcohol and marijuana users compared to controls [35,36], and expected negative associations with neurocognitive performance [36]. Research has also shown some subtle differences between adolescent alcohol and combined alcohol and marijuana users, despite overall poorer white matter integrity in these two groups of teens compared to non-users after early initiation and repeated regular use [36,37,38,39]; such findings highlight the deleterious effects of alcohol on white matter tissue integrity independent from marijuana. Recently, data from our laboratory found that poorer white matter integrity predicted increased future substance use and aggressive/delinquent behaviors, which suggests that some of these imaging biomarkers may have clinical utility in predicting future outcomes [37]. 

In terms of clarifying if white matter abnormalities are related to dose-dependent effects of alcohol and/or marijuana, compared to pre-existing differences driving these behaviors, there are still few investigations that have been able to help address this question. Bava and colleagues (2012) found that the cumulative use of alcohol predicted future white matter health, independent of white matter health pre-initiation, providing some supporting evidence of the detrimental effects of alcohol regardless of potential pre-exiting differences [38]. 

Although cortical thickness differences have been noted prior to initiation of cannabis use [25], few, if any, studies have examined pre-existing white matter integrity in adolescent marijuana users and changes with increased use. This study examined the white matter and neurocognitive trajectories of adolescents that increased alcohol use compared to those that increased alcohol use *and* initiated marijuana use. We hypothesized that there would be no differences in white matter architecture or neurocognitive performance between teens prior to initiation (ages 16–18); however teens that later transitioned to heavy alcohol and marijuana use would show poorer cognition and white matter health than those using alcohol only by young adulthood (ages 19–22). 

## 2. Results and Discussion

### 2.1. Demographics

**Figure 1:**
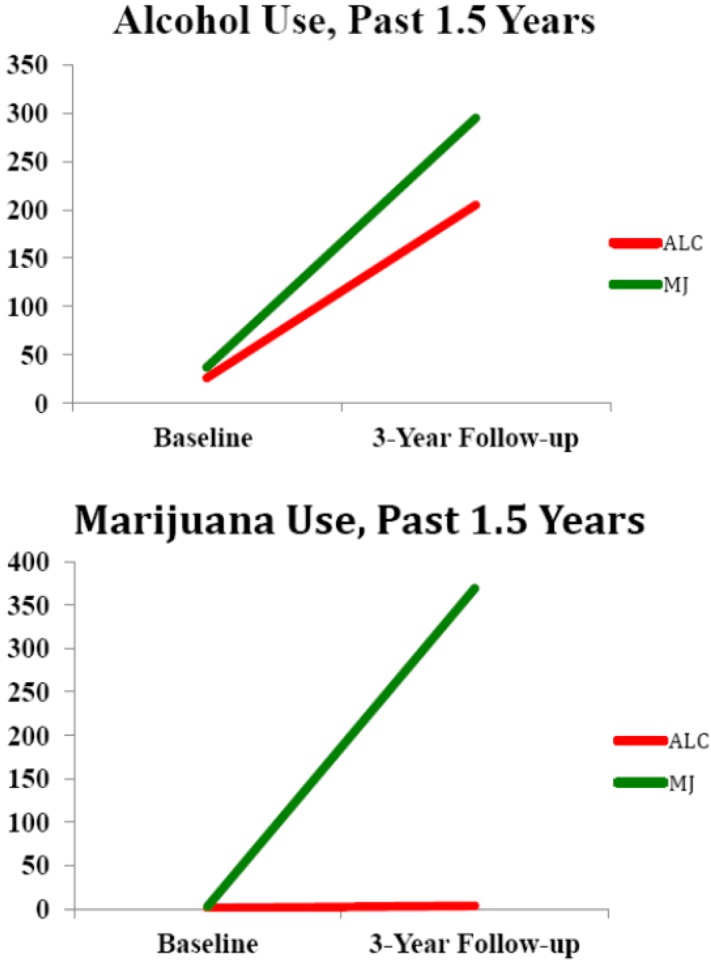
Participant alcohol and marijuana use at baseline and 3-year follow up (*n* = 16) for substance initiators (average marijuana use episodes at baseline, ALC = 1.0; MJ = 3.6 and follow-up, ALC = 3.0; MJ = 369.1)

Groups did not differ on age, gender, family history, or socioeconomic status at baseline (*p* > 0.05). Groups differed on externalizing and internalizing symptoms at baseline, and externalizing symptoms and state anxiety symptoms at 3-year follow-up (*p* < 0.05). Although not significant (*p* > 0.05), marijuana initiators (MJ) reported more other drug use episodes compared to alcohol initiators (ALC) at 3-year follow-up (see Table 1 and Figure 1). 

**Table 1:** Demographic characteristics at 3-year follow-up unless otherwise noted.

	Alcohol initiators (ALC) (*n* = 8) Mean (SD)	Marijuana initiators (MJ) (*n* = 8) Mean (SD)
Age (SD; range) at follow-up	21.2 (19.9–22.1)	20.6 (19.7–22.7)
Age (SD; range) at baseline	18.2 (0.7; 16.9–18.8)	17.5 (0.8; 16.6–19.0)
% Caucasian	75%	63%
GPA at 3-year follow-up	3.3 (0.6)	3.1 (0.3)
Household Income at 3-year follow-up	105.9K (57.3)	163.3K (124.7)
Family history alcohol use disorder	38%	50%
Family history substance use disorder	25%	62%
Alcohol, lifetime use days at baseline (SD: range)	26.3 (20.5; 3–60)	37.1 (33.2; 0–85)
Alcohol, days use over follow-up (SD; range)	205.9 (74.7; 131–312)	295.0 (197.0; 100–625)
Binge Drinking Episodes over follow-up	88.1 (71.2)	156.3 (157.4)
Marijuana, lifetime use days at baseline (SD; range)	1.0 (2.1; 0–6)	3.6 (3.5; 0–9)
Marijuana, days use over follow-up (SD; range) *	3.0 (3.7; 0–10)	369.1 (168.3; 33–540)
Other drug use, lifetime use at baseline (SD; range)	1.0 (0.7; 0.0–2.0)	1.9 (4.5; 0.0–13.0)
Other drug use, days use over follow-up (SD; range)	1.0 (0.3; 0.0–2.0)	20.0 (33.1; 0.0–99.0) ^†^
Beck Depression Inventory at baseline	1.0 (1.0)	4.6 (3.7)
Beck Depression Inventory at 3-year follow-up	1.0 (1.6)	5.5 (7.5)
State Trait Anxiety Inventory at baseline	24.3 (2.6)	29.1 (13.2)
State Trait Anxiety Inventory at 3-year follow-up *	21.3 (2.4)	29.8 (8.1)
Vocabulary *T*-score at baseline	62.4 (9.0)	57.5 (8.6)
CBCL Externalizing *T*-score at baseline *	38.3 (7.7)	52.1 (9.8)
ASR Externalizing *T*-score at 3-year follow-up *	41.3 (12.8)	59.4 (10.7)
CBCL Internalizing *T*-score at baseline *	41.0 (8.3)	50.4 (8.1)
ASR Internalizing *T*-score at 3-year follow-up	38.5 (11.3)	49.0 (15.5)

* *p* < 0.05; ^†^ One sub with ninety-nine episodes, otherwise range (0–27).

### 2.2. White Matter Integrity over Time

**Figure 2:**
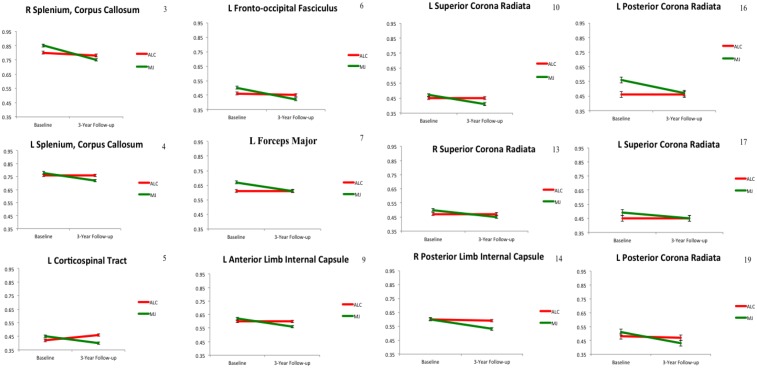
Group by time interactions for WM integrity showing *decreasing* FA in MJ and *no* effect of time in ALC (*n* = 16, *p* < 0.01); numbers corresponds to cluster order.

**Figure 3:**
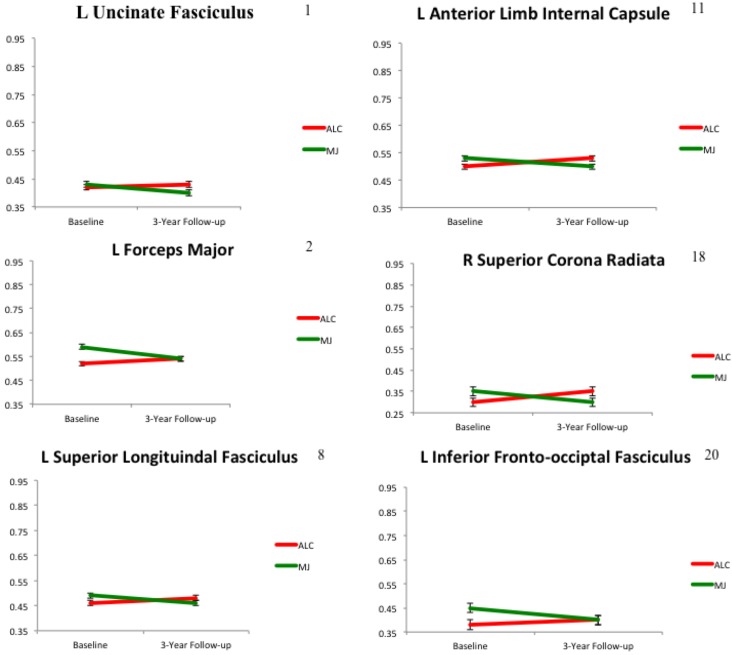
Group by time interactions for WM integrity showing *increasing* FA in ALC and *decreasing* FA in MJ (*n* = 16, *p* < 0.01); numbers correspond to cluster order.

Repeated measures ANOVA revealed 20 clusters in which a significant group by time interaction effect was found in association, projection, and interhemispheric fiber tracts, *F*(1,14) = 16.7–67.4, *p* < 0.01. In 12 clusters, we saw *no effect* of time for ALC, but FA *decreases* from baseline to 3-year follow-up for MJ (see Figure 2 for regions). In 6 clusters, we saw FA *increasing* for ALC and *decreasing* for MJ (see Figure 3 for regions). In one cluster we observed increasing FA in ALC with no effect of time for MJ (left forceps minor), and in one cluster we observed FA decreasing for both ALC and MJ, with MJ decreasing at a steeper rate (right splenium corpus callosum). 

Analysis were re-examined controlling for internalizing, externalizing, binge drinking episodes, and other drug use episodes, and all findings remained significant (*p* < 0.01). Binge drinking episodes were not found to be a unique predictor of change in FA from baseline to follow-up in the larger sample (*p* > 0.05).

### 2.3. Neurocognitive Functioning over Time

**Figure 4:**
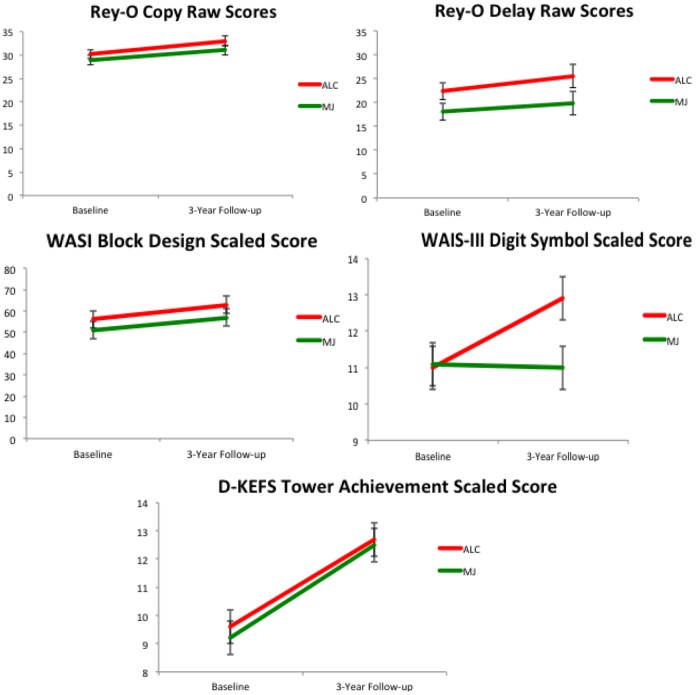
Main effects of time (Rey, Block Design, Tower) and group by time interactions (Digit Symbol) for neuropsychological performance over three years (*n* = 16), norm-based scaled scores were corrected for age.

Time and group by time interaction effects were observed on five neurocognitive tests. A main effect of time was found on the Rey-O Copy, *F*(1,14) = 11.9, *p* < 0.01, Rey-O Delay *F*(1.14) = 5.5, *p* = 0.03, WASI Block Design subtest scaled score *F*(1,14) = 18.2, *p* < 0.01, and DKEFS Tower Test total achievement scaled score *F*(1,14) = 17.0, *p* < 0.01 (better performance over time). A group by time interaction, *F*(1,14) = 8.1, *p* = 0.01, was found on the WAIS-III Digit Symbol subtest scaled score, as increasing performance over time was observed in ALC; however, no change was observed in MJ (Figure 4). 

#### Neurocognitive Correlates with White Matter

**Figure 5:**
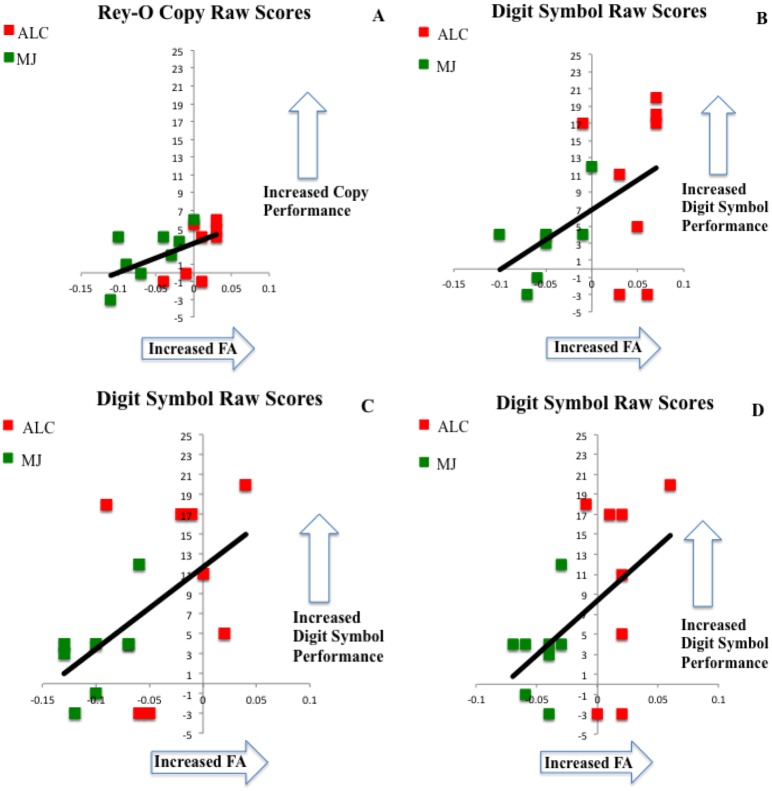
Bivariate relationships between change in FA and change in neuropsychological performance (baseline subtracted from 3-year follow-up for both FA and neuropsychological measures); (**A**) FA in right superior corona radiata, (**B**) FA in right superior corona radiata, (**C**) FA in the right splenium of the corpus callosum, (**D**) FA in left forceps major.

Bivariate correlations exploring change in white matter integrity (FA) between 3-year follow-up and baseline and change in neuropsychological performance between 3-year follow-up and baseline were evaluated. A positive relationship was found between increased FA in the right superior corona radiata (*r* = 0.52, *p* = 0.03) and improved performance on the Rey-O Figure Copy. Increased FA in the right superior corona radiata (*r* = 0.50, *p* = 0.04), right splenium of the corpus callosum (*r* = 0.55, *p* = 0.03), and left forceps major (*r* = 0.52, *p* = 0.04) were also found to be related to improved performance on the Digit Symbol subtest. Relationships were not moderated by group status (see Figure 5). No significant correlations were found between substance use severity at follow-up and neuropsychological performance.

### 2.4. Key Findings and Limitations

The aim of this preliminary study was to look at pre- and post white matter differences in teens after initiation of alcohol or alcohol *and* marijuana use in later adolescence (ages 16–22). We suspected teens that initiated alcohol and marijuana use would show decreasing FA values over time, as compared to those who only initiated alcohol use. In the vast majority of clusters, we found *decreasing* FA in alcohol and marijuana initiators and no effect of time in alcohol only initiators. A second group of clusters revealed *increasing* FA in alcohol only initiators (as would be expected in adolescent brain development) and *decreasing* FA in alcohol and marijuana initiators. Overall, we found that teens that initiated both heavy alcohol use *and* marijuana use demonstrated evidence of declining white matter integrity in association, projection, and corpus callosum fiber tracts, areas showing continued development throughout young adulthood [40]. We saw the inverse relationship for teens that initiated alcohol use only in late adolescence/early adulthood, as there was evidence for ongoing improvement in white matter integrity or limited change over time. Notably, in several clusters, marijuana initiators had average FA values equal to or significantly greater than alcohol only initiators, supporting evidence for marijuana-related toxicity, as opposed to pre-existing morphological tissue differences driving these relationships. Further, we saw evidence of improved white matter integrity (*i.e.*, increased FA) linked to enhanced neurocognitive performance, along with improvement in neurocognitive performance over time in both groups of teens. It is possible that the bivariate relationships between change scores is largely driven by the improved performance on Digit Symbol over time in the ALC group; however the associations were not found to depend on group status. 

While previous investigations in our laboratory have identified poorer white matter integrity in adolescent alcohol users compared to controls [17,36], and in some cases compared to those with marijuana use histories [40], this study is unique in that we identified a subset of teens at an initial time point with minimal use histories and evaluated them 3-years later, after initiation of either heavy alcohol use or heavy alcohol *and* marijuana use; previous work in our laboratory has predominately looked at between-group differences after initiation, as most teens enter the study and remain within their substance use patterns. In a recent study under review, we have observed poorer tract integrity in those adolescents with three or more years of regular substance use and younger age at initiation, including both alcohol and combined alcohol and marijuana use. 

Elucidating pre-existing differences and changes associated with alcohol and marijuana use has been difficult. While pre-existing differences (for example, personality factors, or non-white matter related brain discrepancies) may still be driving the reported relationships in this investigation, there is strong support for a deleterious effect of combined alcohol and marijuana use on white matter health in those teens that transitioned to heavy use after their baseline assessment. Furthermore, the timing at which the teens initiated their heavier substance use differs from our previous white matter investigations. For example, in the vast majority of our investigations [17,35,40], teens were recruited into the study having already initiated alcohol and/or marijuana (prior to age 17), however in this sample, teens initiated heavier use around age 18 or older (on average), which is likely contributing to some discrepancies in findings (e.g., more subtle alcohol effects in the present investigation may be related to initiation of alcohol at an older age). 

Few prospective studies have looked at pre-existing differences or subsequent brain changes in marijuana users prior to or after initiation of use. Cheetham and colleagues (2012) suggest evidence of pre-existing macrostructural brain differences, in that smaller orbitofrontal cortex volume predicted initiation of marijuana use by age 16. In our lab we have seen poorer white matter integrity predict increased substance use over an 18-month follow-up interval [37]. It is possible that neural circuits underlying particular brain regions important for cognitive control and reward may lead to vulnerabilities, which subsequently lead to marijuana use [25,37]. A recent investigation suggests a decline in cognitive functioning with persistent cannabis use. This longitudinal study found declines from premorbid functioning (assessed at age 13) after initiation of persistent cannabis use (age 38), on WAIS-IV IQ performance, and adolescent-onset users showed greater decline [22]. Studies such as these, taken together with the present findings, underscore two important points. First, it is likely that there are pre-existing neural vulnerabilities to initiate substance use and continue consumption of marijuana in some teens. Second, despite these potential existing differences, we are also likely to observe a negative effect of marijuana and alcohol use on brain tissue development whether pre-existing differences are present in the individual and/or sample. Changes in tissue development are likely related to cognitive alterations in cognitive functioning, even if changes are subtle. 

While there are limited prospective white matter studies in adolescent marijuana users prior to initiation, cross sectional studies outside of our laboratory have also largely shown differences in adolescent marijuana users compared to non-using controls teens, despite some limited evidence to the contrary [41]. In 2009, Ashtari and colleagues [42] observed poor tract integrity in adolescent marijuana users. Similarly, in 2010, Yucel and colleagues [43] also found poorer white matter integrity in marijuana users when compared to controls. Gray matter alterations have been observed in several cross sectional investigations as well. For example, we have seen some discrepancies in prefrontal cortex, amygdala, and cerebellum volume in adolescent cannabis users compared to controls in our studies [44,45,46]. Other laboratories report dose-dependent relationships with temporal lobe structures such as hippocampus and amygdala [47] and alterations in cortical thickness [27] in frontal and parietal regions. Alterations in microstructural tissue integrity and macrostructural volume suggest a neurotoxic effect of marijuana on adolescent neurodevelopmental trajectories. Cannabinoid receptors are believed to modulate neurotransmission and may influence genetic expression of neurodevelopment [48], which may underlie many of the neurocognitive and structural neuroimaging differences observed in human subjects research with adolescent marijuana users. Preclinical evidence suggests cognitive/behavioral and social changes after exposure to Δ^9^-tetrahydrocannbinol (THC), as well as other cannabinoid agonists in adolescent rats [49,50].

While the strengths of this investigation include the prospective design and limited lifetime substance use episodes besides alcohol and marijuana, there are several limitations that must be noted. The sample size is small and multiple comparison corrections were less stringent and therefore replication of this preliminary work is important. Further, while alcohol use did not statistically differ between the groups (e.g., lifetime use, binge drinking) or predict change in white matter integrity, the marijuana users do report more binge drinking episodes and it is possible frequency and severity of alcohol use (*vs.* marijuana-related toxicity) contribute to the between group differences reported in this study. Future work should also focus on identifying and following a representative group of adolescents ages 19–22 with minimal alcohol and marijuana use to better disentangle the effects of these substances; however identifying and recruiting adolescents ages 19–22 who do not transition to some degree of heavy episodic drinking is challenging given the prevalence of alcohol use during this developmental window. Of course, self-report measures (e.g., lifetime alcohol use, marijuana use) can be limited and may impact statistical estimates. 

## 3. Experimental Section

### 3.1. Participants

Participants in this study were adolescents ages 16–18 who were originally recruited as controls in an ongoing longitudinal study of adolescent marijuana and alcohol users (R01 DA021182), but who initiated heavy alcohol and/or marijuana use over the follow-up period (3 years post-baseline). At baseline, all participants (*n* = 16) had minimal substance use (see Table 1 and Figure 1). At the 3-year follow-up they were classified into two groups, those who reported very limited marijuana use (≤10 use episodes each visit), minimal alcohol use at baseline, and significantly increased alcohol use at 3-year follow-up (ALC, *n* = 8), and those who reported minimal marijuana use at baseline, minimal alcohol use at baseline, and significantly increased alcohol *and marijuana* use at 3-year follow-up (MJ, *n* = 8). 

Adolescents were recruited from local schools. A parent or guardian was required to provide consent to participate in the study at baseline, and adolescents were required to provide assent in accordance with the University of California, San Diego Research Protections Program procedures. Exclusionary criteria were: history of a lifetime DSM-IV Axis I disorder (other than cannabis or alcohol abuse or dependence), history of learning disability, history of neurological disorder or head trauma with loss of consciousness >2 min, history of a serious physical health problem, complicated or premature birth; uncorrectable sensory impairments; left handedness; MRI contraindications, and use of psychoactive medications at project intake. Weekly toxicology screening was performed for four weeks prior to neuropsychological testing and neuroimaging scan session at both time points to confirm abstinence from marijuana prior to visit.

### 3.2. Procedures

At the baseline and 3-year follow-up, teens received a detailed substance use and mental health interview, diffusion tensor imaging session, and a comprehensive neuropsychological evaluation. Parents were also administered detail interviews to corroborate both demographic, psychosocial functioning, and substance use behaviors.

#### 3.2.1. Substance Use and Mental Health Assessment

Substance use was assessed using the Customary Drinking and Drug Use Record (CDDR) [51]. This measure provided characterization of lifetime (baseline) and past 1.5-year use of alcohol, marijuana, nicotine, and eight other classes of illicit drugs at each appointment; DSM-IV abuse and dependence criteria were also collected. The Child Behavior Checklist (baseline) and corresponding Adult Self Report (3-year follow-up) was used to identify externalizing and internalizing symptoms experienced in the 6-months prior to appointment [52]. The Beck Depression Inventory and State Trait Anxiety Inventory [53,54] were used to assess depressive symptoms and state anxiety. Family history of substance use and psychiatry disorders was assessed with the Family History Assessment Module [55]. 

#### 3.2.2. Neuropsychological Testing

Performance on approximately twenty standardized neuropsychological tests examining the domains of complex attention, processing speed, verbal memory, visuospatial functioning, and executive functioning were examined pre- and post alcohol and marijuana initiation. Measures include: (1) *complex attention*: California Verbal Learning Test-II Trial 1 and Trial 1–5 Total Recall (CVLT-II) [56], Wechsler Adult Intelligence Scale-Third Edition (WAIS-III) [57], Digit Symbol, Arithmetic, and Digit Span Backward subtests; Paced Auditory Serial Addition Test (PASAT) [58]; (2) *processing speed*: Delis-Kaplan Executive Function System (D-KEFS) Trail Making Test Number Sequencing, Letter Sequencing, and Motor Speed subtests [59]; (3) *verbal memory*: Wechsler Memory Scale-Third Edition (WMS-III) [60], Logical Memory I, II, and Recognition; CVLT-II Short and Long Delay Free Recall, CVLT-II Recognition [56]; (4) *visuospatial functioning*: Rey-Osterrieth Complex Figure Copy and Delay Accuracy (Rey-O) [61], Wechsler Abbreviated Scale of Intelligence Block Design subtest (WASI) [62]; and (5) *executive functioning*: D-KEFS Trial Making Test Number-Letter Switching, Tower Test Achievement, and Verbal Letter Fluency subtests [59]. 

#### 3.2.3. Diffusion Tensor Imaging Acquisition and Processing

DTI scans at baseline and 3-year follow-up were acquired at the University of California, Center for Functional MRI (CFMRI) on a General Electric 3.0T magnet using an 8-channel gradient head coil. Diffusion was encoded through a single-shot dual spin echo excitation along 15 diffusion directions with b = 2000 s/mm^2^ and 4 averages in addition to the normalization image with no diffusion encoding (b = 0). Image parameters for the DTI sequence were: TE = 93.4 ms, TR = 12,400 ms, FOV = 24 cm, 36 contiguous slices, slice thickness = 3.0 mm, and image matrix = 128 × 128. Two field maps (TE/TR = 3.8/1000 ms) were collected and applied to correct for signal loss and inhomogenities in the magnetic field [63].

Image processing was conducted using software from the Oxford Centre for Functional Magnetic Resonance Imaging of the Brain software library (FSL) [64]. Pre-alignment processing steps included a six-degree of freedom affine registration for head motion and eddy current distortion (FLIRT-FMRIB’s Linear Registration Tool) [65] and correction for field distortions using PRELUDE (Phase Region Expanding Labeler for Unwrapping Discrete Estimates) [66] and FUGUE (FMRIB’s Utility for Geometrically Unwarping EPIs) [67]. Pre-processed images were then subjected to tensor decomposition to derive fractional anisotropy (FA) estimates. FMRIB’s Diffusion Toolbox [68] was used to calculate diffusion estimates for each subject. Tract-based spatial statistics (TBSS) identified white matter tracts common to all participants at both baseline and 3-year follow-up (for further description see [35,36,40]). Individual FA maps were registered to a standard template in MNI-152 space and averaged across participants to create a mean FA image. A white matter skeleton was then derived representing centers of all tracts common to all participants. To minimize partial-volume effects, values were thresholded at FA > 0.2. FA data from each subject was projected onto the derived skeleton and resulting data formed the basis of voxelwise cross-subject statistical comparisons [69].

### 3.3. Data Analyses

#### 3.3.1. Demographic Comparisons.

One-way analysis of variance (ANOVA) and Chi-square tests examined between group differences on several demographic characteristics (e.g., age, ethnicity, mental health functioning) at baseline and 3-year follow-up. 

#### 3.3.2. DTI Statistical Analyses.

Whole brain repeated-measures analysis of variance were carried out (Analysis of Functional NeuroImages (AFNI, *3dANOVA3*) [70] to identify between-group and within-subject differences across all participants at baseline and 3-year follow-up. Main effects of time, group, and their interaction were specified; it was determined *a priori* that if the interaction was significant, only clusters showing significant interaction effects would be examined in follow-up analyses. Multiple comparisons were corrected with voxel probability and cluster size thresholding using Monte Carlo simulations. Only clusters ≥9 µL (9 contiguous 1 × 1 × 1 mm voxels) with an individual voxel effect of α < 0.01 were interpreted, yielding a brain-wise α < 0.05 of finding such a cluster under the null hypothesis.

#### 3.3.3. Neuropsychological Performance, White Matter Correlates, and Substance Use Severity

Repeated-measures ANOVA was used to examine neurocognitive trajectories at baseline and 3-year follow-up for each cognitive measure listed above. Main effect of time, group, and their interaction was specified. Change scores (baseline subtracted from 3-year follow-up) were computed for all 20 DTI clusters found to be significant and all neuropsychological measures (raw scores). Bivariate correlations between DTI change scores and changes in neuropsychological performance were examined. Similarly, bivariate correlations were explored between substance use severity (e.g., marijuana, alcohol, and binge drinking episodes over past 1.5 years) reported at 3-year follow-up and neuropsychological performance at follow-up.

## 4. Conclusions

In conclusion, this prospective study found decreased white matter integrity for teens that initiated heavy alcohol *and* marijuana use three years after a baseline assessment. Limited findings were identified for those that initiated alcohol use only in late adolescence/early adulthood. The data suggests a deleterious effect of marijuana use on white matter health in the context of heavy alcohol use. While previous studies from our laboratory also find poorer white matter integrity in teens who report alcohol use during earlier adolescence (prior to age 17), this study finds a lesser impact of alcohol use on white matter tissue integrity for those that initiate alcohol use only at a later age (over 18 *vs.* prior to age 17). We also observed positive associations between increased white matter health and better neurocognitive functioning in the combined sample. Future follow-up investigations will focus on replication with larger sample sizes, and explore the role of gender (hormones) and genetics (family history).

Importantly, combining multimodal neuroimaging will help to examine the relationships between white matter integrity, gray matter volume, and cortical thickness in relation to adolescent substance use, prior to and after escalation of use. We hope to identify brain changes that are the result of increased substance use in order to appropriately develop cognitive interventions. Neuroimaging biomarkers for predicting youth at increased vulnerability for substance use disorders and other risk taking behaviors is critical for prevention of functional decline and future development of mental health disorders that typically arise during young adulthood.
